# Molecular alterations of cancer cell and tumour microenvironment in metastatic gastric cancer

**DOI:** 10.1038/s41388-018-0341-x

**Published:** 2018-05-23

**Authors:** Weilin Li, Jennifer Mun-Kar Ng, Chi Chun Wong, Enders Kwok Wai Ng, Jun Yu

**Affiliations:** 1 Department of Surgery, The Chinese University of Hong Kong, Hong Kong, Hong Kong; 2 Institute of Digestive Disease, Department of Medicine and Therapeutics, State Key Laboratory of Digestive Disease, Li Ka Shing Institute of Health Sciences, The Chinese University of Hong Kong, Hong Kong, Hong Kong

## Abstract

The term metastasis is widely used to describe the endpoint of the process by which tumour cells spread from the primary location to an anatomically distant site. Achieving successful dissemination is dependent not only on the molecular alterations of the cancer cells themselves, but also on the microenvironment through which they encounter. Here, we reviewed the molecular alterations of metastatic gastric cancer (GC) as it reflects a large proportion of GC patients currently seen in clinic. We hope that further exploration and understanding of the multistep metastatic cascade will yield novel therapeutic targets that will lead to better patient outcomes.

## Introduction

Gastric cancer (GC) is the fourth most common cancer and second leading cause of cancer-related deaths worldwide [1]. Over 70% of GC cases (~677,000 per annum) occur in the developing regions, mainly in Asia, Central and Eastern Europe and Latin America [2–4]. Despite improvements in GC incidence and mortality over the last decade, the disease burden still remains high. The majority of patients present with clinically advanced disease such that curative surgical resection is no longer possible and current therapeutics are poor at controlling the progression of metastatic disease. More worryingly, there are suggestions that advancements in GC treatment are likely to be surpassed by other diseases, consequently some foresee that GC is on a rising trend as a leading cause of death worldwide [5].

Metastasis is the main cause of cancer mortality (>90%) and a critical step that hampers the development of anti-cancer therapy due to its systemic nature and resistance to existing therapeutic drugs [6, 7]. Metastasis of gastric adenocarcinoma is no exception. It represents a multistep biological cascade that ultimately leads to widespread dissemination of carcinoma cells in various tissue sites [6, 8, 9]. In this review, we take you step-by-step through the GC metastatic cascade and the current understanding of the spectrum of molecular alterations involved. We look forward to this update being a guide for future research, and at the same time, highlighting its potential for translation into therapeutic strategies.

## GC metastasis cascade

**Fig. 1:**
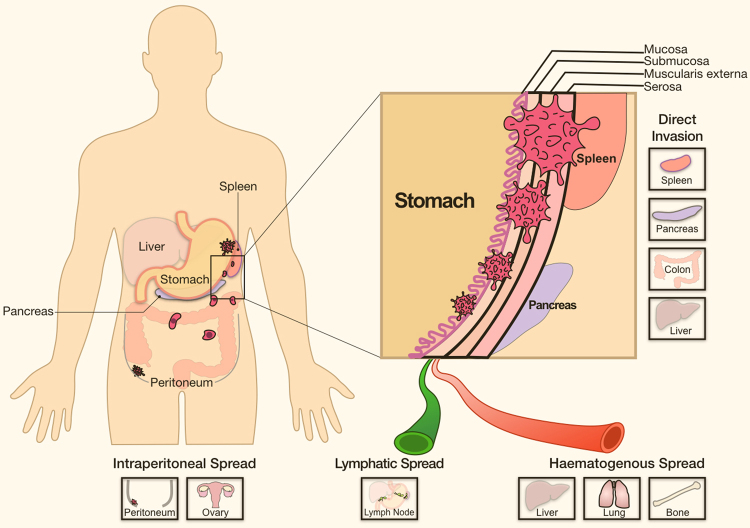
Metastatic routes and sites in gastric cancer. Major routes of distant metastasis in gastric cancer: intraperitoneal, lymphatic and haematogenous spread, and direct invasion into neighbouring organs. Common sites of metastases: spleen, pancreas, colon, liver, peritoneum, ovary, lymph nodes, lung and bone

GC most commonly metastasises to the liver, peritoneum, lung, bone and lymph nodes [10] either through direct invasion or more distant seeding via the blood, lymphatic system and intraperitoneal spread. Notwithstanding these differences, they share the following series of sequential and interrelated events: (1) local invasion into the surrounding tumour-associated stroma, (2) intravasation into the haematopoietic or lymphatic systems, or intraperitoneal spread, (3) survival in vasculature transition or intraperitoneal fluid circulation, (4) extravasation into 'fertile soil' at distant organs with pre-metastatic niches and (5) colonisation and proliferation to form detectable metastases (Fig. 1) [7, 11–13]. These cellular events are normally kept in check under the orchestration of both intrinsic and extrinsic molecular pathways; however, aberrant molecular alterations allow the transformation of nascent tumour cells to highly invasive malignancies, which further lead to incurable metastatic disease with systemic spread and therapeutic resistance [6].

## Local invasion into surrounding tumour-associated stromal microenvironment

Local invasion occurs when tumour cells no longer obey the delineation of the basement membrane (BM), and the invasive front infiltrates the neighbouring tumour-associated stroma and surrounding normal tissues. Three major players facilitate this process: epithelial–mesenchymal transition (EMT), matrix metalloproteinases (MMPs) and the stromal environment, within which alterations and interactions amongst various molecular processes determine the tumour cells’ invasive propensity [14].

## EMT

**Fig. 2:**
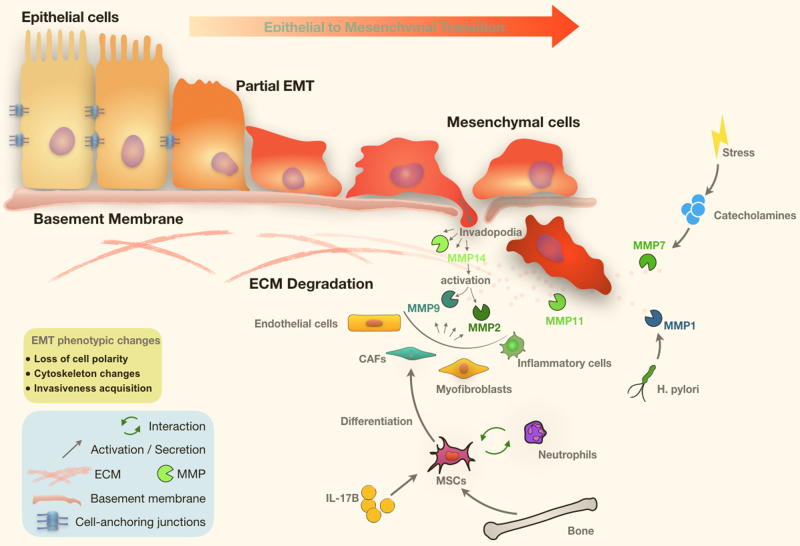
Epithelial–mesenchymal transition and tumour–stromal interactions in gastric cancer. Main phenotypic changes of EMT in gastric cancer include loss of cell polarity, degradation of cell-anchoring junctions, cytoskeleton changes, acquisition of invasiveness and ultimately degradation of basement membrane. Interactions within between key components of the stromal environment. EMT epithelial–mesenchymal transition, ECM extracellular matrix, CAF cancer-associated fibroblast, MSC mesenchymal stem cell, MMP matrix metalloproteinase

EMT describes the dissociation of tightly knitted epithelial cells and subsequent transdifferentiation into motile and invasive mesenchymal cells [15]. In the mesenchymal cell state, these cancer cells possess novel ability to invade into the surrounding microenvironment. Thus, EMT is considered to be the crucial step in the initiation of local invasion, and hence subsequent dissemination [14]. The transition involves relocalisation, dissolution and degradation of adherens junctions, subapical tight junctions, desmosomes and gap junctions between epithelial cells, ultimately leading to the loss of cell polarity and cytoskeleton changes [14–16]. As EMT becomes more established, mesenchymal phenotypes become more prominent, and the cells start to possess the ability to degrade extracellular matrix (ECM) proteins (Fig. 2) [15].

**Fig. 3:**
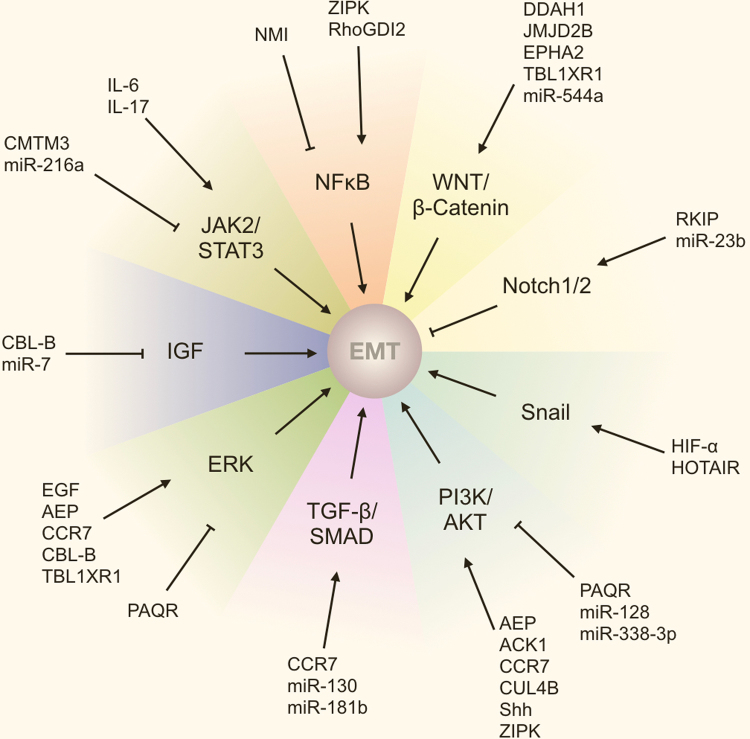
Molecular mechanisms of EMT in gastric cancer. Major signalling pathways that regulate EMT in gastric cancer. PI3K/AKT, WNT/β-Catenin, ERK, TGF-β/SMAD and Snail signalling pathways promote EMT; Notch1/2 inhibits EMT in gastric cancer

In GC, a number of signalling pathways have been found to regulate EMT, with the PI3K/AKT, MEK/ERK and WNT/β-Catenin pathways taking leading roles (Fig. 3). Transcription factors (TFs) and microRNAs, as described below, are the primary modulators. Although either can act independently, there is often some cross-modulation and interdependence that provides further complexity to their role in the regulation of signalling pathways.

### Transcription factors

EMT is tightly regulated by TFs. Apart from the prominent TFs, such as Slug [17], Snail [18], Twist1/2 [19, 20], FOXQ1 [21] and ZEB1/2 [22–25] in GC, there are emerging novel TFs that have also been found to regulate EMT. For example, runt-related TF 3 (RUNX3), which has a role in suppressing EMT through the TGF-β-activated SMAD pathway, has been observed to be frequently downregulated [26, 27]. Similarly, there is loss of RUNX3-dependent miR-30a activation which normally inhibits vimentin expression and EMT [28]. In addition, a study based on array profiling identified significant upregulation of serum response factor (SRF) in metastatic GC cells. SRF functions to promote EMT through miR-199a-5p-mediated decrease in E-cadherin expression [29]. Also of interest, HOXB9 has been shown to halt GC progression. Studies have shown that restoration of HOXB9 expression in GC cells led to inhibited invasion and migration, at the same time stimulated the reversal of EMT process [30].

### microRNAs

EMT can also be modulated by microRNAs [28]. For example, miR-544a induces EMT, as shown by the decreased expression of E-cadherin, APC2 and AXIN2, which stabilises the nuclear import of β-catenin and activates Wnt signalling to promote cell invasiveness in GC cell lines [31]. miR-2392 inhibits EMT through downregulating TFs, such as Slug and Twist1, in GC cells [32]. miR-223 promotes GC cell invasion and resistance to cisplatin by targeting FBXW7 [33, 34]. Conversely, miR-338-3p suppresses EMT through downregulating ZEB2, a TF that plays a vital role in promoting EMT in GC [25]. miR-506 suppresses EMT directly and its low expression is correlated with poor prognosis which indicates that it can serve as an independent prognosis fact in GC patients [27, 35]. Therefore, microRNAs can also be utilised as a potential therapeutic target for blocking EMT progression. For example, AC1MMYR2, a specific small-molecular inhibitor that can block the maturation of pre-miR-21 to miR-21, has been shown to reverse EMT and eventually lead to the suppression of GC cell metastasis [36].

### Other molecules

In addition to TFs and microRNAs, EMT can be regulated by many other endogenous molecules. For example, Jumonji domain-containing protein 2B (JMJD2B) and erythropoietin-producing hepatocellular A2 (EphA2) which belongs to hydroxylase superfamily and protein-tyrosine kinase family, respectively, have both been suggested to induce EMT via the Wnt/β-catenin signalling pathway and further stimulate GC development and metastasis [37, 38]. Another example is melatonin, which a recent in vitro study revealed suppression of EMT in GC cells via the induction of endoplasmic reticulum stress and inhibition of β-catenin activity. Melatonin therapy decreased peritoneal dissemination in mice [39]. A more comprehensive list of the genes and microRNAs involved in regulating EMT are listed in Tables 1 and 2.

**Table 1:** Molecular alterations that promote EMT

Molecular alterations	Signalling pathways	References
ACh	M3R/AMPK/MACC1	[155]
ACK1	AKT/POU2F1/ECD	[156, 157]
AEP	AKT/MARK	[158]
CCR7	TGFβ/ERK/PI3K/Snail	[159–161]
CUL4A	Hippo	[162]
CUL4B	PI3K/AKT	[163]
ECM1	ITGB4/FAK/SOX2/HIF-1α	[164]
EGF	Arf6-ERK	[22, 165–167]
EphA2	WNT/β-Catenin	[38, 168, 169]
FOXK1	C-jun	[170]
FOXM1	–	[171]
FOXO3a	–	[172]
HIF-1α	Snail	[173]
HOTAIR	HGF/C­Met/Snail	[174]
IFITM2	IGF1/IGF1R/STAT3	[175]
IL-6	JAK2/STAT3	[176]
IL-17	STAT3	[177]
JMJD2B	WNT/β-Catenin	[37, 178, 179]
MICAL2	–	[180]
Orai1, STIM1	–	[181]
Rab11-FIP2	–	[182]
RBP2	TGF­β1/Smad3	[183]
RhoGDI2	NFκB/Snail	[18, 184, 185]
SALL4	–	[186]
SENP3	–	[187]
Shh	PI3K/AKT	[188]
SPOCK1	–	[17, 189, 190]
SRF	–	[29, 191]
TBL1XR1	β­catenin/MMP7/EGFR/ERK	[192]
TMPRSS4	–	[193]
ZIPK	AKT/IκB/NF­κB	[194]
miR-21	–	[36]
miR-130	TGF­β	[195]
miR-181a-5p	MAKP	[196]
miR-181b	TGF­β/SMAD2/3/4	[197]
miR-363	–	[198]
miR-421	–	[199]
miR-544a	WNT	[31]
miR-940	–	[200]

**Table 2:** Molecular alterations that suppress EMT

Molecular alterations	Signalling pathways	References
ARID1A	–	[201]
CBL-B	AKT/ERK	[202]
CMTM3	STAT3/Twist1/EMT	[203]
DDAH1	WNT/β-Catenin	[204]
FBXL5	–	[205]
FBXW7	RhoA/p53	[33, 34, 206–211]
HOXB9	–	[30, 212, 213]
NMI	NFκB/p65	[214]
PAQR3	Raf/MAPK PI3K/AKT	[19, 215, 216]
PDK1	–	[217]
PPARγ	–	[218]
Rap1GAP	–	[219]
RKIP	Notch1	[220–222]
TOP1MT	–	[223]
miR-BART6-3p	–	[224]
miR-7	IGF	[225]
miR-22	–	[69]
miR-23b	Notch2	[226]
miR-128	PI3K/AKT	[227]
miR-143, miR-145	–	[228]
miR-200b	–	[229]
miR-216a	JAK2/STAT3	[230]
miR-338-3p	MET/AKT/PTEN	[25, 231–233]
miR-551b	–	[234]
miR-1271	–	[21]
miR-2392	–	[32]

## MMP

The BM is an important regulator of cellular behaviour in addition to its passive role in supporting surrounding tissues [40]. In cancer, BM functions as a mechanical barrier that prohibits cancer cells from penetrating the neighbouring stroma [41]. MMPs are proteolytic enzymes with a physiological role in degrading ECM proteins. However, dysregulation of MMPs, as seen in cancer cells, lead to uncontrolled proteolytic activity, tissue remodelling and disproportionate degradation of BM, thereby granting tumour cells stromal access [42, 43]. MMPs are upregulated in nearly all cancers, and their increased expressivity is generally associated with a poorer prognosis. Accumulating evidence has highlighted the role of MMPs in lymph node metastasis, peritoneal metastasis and distant metastasis [44–49].

### MMP-1

MMP-1 is an interstitial collagenase that plays a role in the degradation of type I collagen (a major ECM component of stomach mucosa) [50–52]. One study reported that *Helicobacter pylori* infection can stimulate the upregulation of MMP-1, which could further enhance the potential of GC metastasis [51].

### MMP-2 and MMP-9

MMP-2 and MMP-9 belong to the family of type IV collagenases or gelatinases. Both have been reported to contribute to vessel invasion and lymph node metastasis in intra-mucosal GC by degrading type IV collagen, which enabled infiltration of lymph capillaries [44, 53]. Certain oncogenic proteins play a role in regulating expression of MMP-2/9 in promoting cell invasion. For example, Bcl-w, which belongs to Bcl-2 protein family, has been demonstrated to induce MMP-2 expression via a sequential activation of PI3K, Akt and Sp1, thereby enhancing cell invasiveness and GC metastasis [54]. Similarly, androgen receptor, which is believed to play vital roles in various types of cancers, has been reported to bind directly to the promoter region of MMP-9, which upregulates MMP-9 expression and in turn promotes GC cell migration and invasion [55]. Furthermore, lipocalin-2, which is regarded as neutrophil gelatinase-associated lipocalin, has recently been shown to prevent auto-degradation of MMP-9. Collectively, both MMP-2 and MMP-9 are highly expressed in GC and their expression is positively associated with the poor survival of GC patients [56].

### MMP-7

MMP-7 (matrilysin) is another MMP that is highly expressed in GC [57, 58]. It is the smallest (molecular weight) member of MMP family but with most efficient ECM-degrading activity on a wide spectrum of matrix substrates, such as proteoglycans, elastin, caseins, laminins, fibronectins, collagens, gelatins, entactins, vitronectins [59–61]. The expression level of MMP-7 at the invasive front of the tumour is relatively higher than the core, which indicates that the upregulation of MMP-7 is associated with cancer aggressiveness in GC [60]. Interestingly, several studies indicate that psychological stress-related changes may be involved in promoting cancer metastasis through stimulating the expression of MMPs [62–64]. For instance, it has been reported that catecholamines, which are stress-inducible hormones responsive to stress, depression or panic, can upregulate MMP-7 expression through AP-1 and STAT3 stimulation [59].

### MMP-11

MMP-11, also known as Stromelysin-3, is distinct from other MMPs as it can only weakly degrade the main components of ECM. Additionally, it needs to be proteolytically cleaved and activated intracellularly by Furin-like serine proteinases prior to its relocalisation to the cell membrane [65, 66]. MMP-11 was first identified as a breast cancer-related gene which was later found to be highly expressed in most metastatic primary tumours as well as in some of their metastases when compared to matched normal tissues. Not only was this evident in GC, it could also be seen in renal, colon and lung cancers [67]. Moreover, one study found that MMP-11 levels were markedly elevated in the serum of GC patients compared with those from healthy subjects, and the enhanced expression of MMP-11 was well associated with metastases in these GC patients [66].

### MMP-14

MMP-14 belongs to one of the six membrane-anchored MMPs, unlike the majority which are secreted proteins [68]. MMP-14 is normally located at the leading edge or invadopodia of a cell, which facilitates the degradation of ECM and guides cells to invade in a specific direction [68]. Additionally, MMP-14 promotes the secretion and activation of pro-MMP-2 and pro-MMP-9 [68, 69]. A recent meta-analysis showed that MMP-14 levels were significantly higher in GC tissues, and the increased MMP-14 expression correlated to higher clinical stage and metastases [70].

While most of the studies thus far focus on cancer cells-derived MMPs, emerging evidence indicate that MMPs (including MMP-2 and MMP-9) can also be secreted by the surrounding stromal cells, such as endothelial cells, fibroblasts, myofibroblasts and inflammatory cells [65]. These findings imply the importance of the tumour microenvironment in GC metastatic progression, as discussed below (Table 3).

**Table 3:** MMPs regulating EMT

MMPs	Functions	Signalling pathways	References
MMP1	Promotes metastasis	–	[51]
MMP2	Promotes angiogenesis, gastric cancer cell survivability and invasiveness; degrades the basement membrane; facilitates permeation into lymphatics	PI3K/Akt/Sp1	[44, 53, 54, 235–237]
MMP7	Unknown. Associated with invasion of the gastric wall, lymph node metastasis, peritoneal metastasis and poor survival of gastric cancer patients	AP-1/STAT3	[46, 59, 60]
MMP9	Promotes cell migration, invasion, lymph node metastasis, distant metastasis and lymphangiogenesis; degrades the basement membrane; facilitates permeation into lymphatics	Sonic Hedgehog (Shh)/Akt	[27, 44, 45, 47–49, 55, 188, 235, 238]
MMP11	Decreases cancer cell death through apoptosis and necrosis; increases proliferation and invasion.	IGF1 pathway	[67, 239, 240]
MMP14	Unknown. Associated with high clinical stage, lymph node metastasis and distant metastasis	–	[68–70]
MT3-MMP	Increases invasiveness	WNT/β-Catenin	[241]

## Stromal environment

When cancer cells reach the surrounding stroma following EMT and BM penetration, the next step involves overcoming the barriers to allow further infiltration. Recent studies revealed that tumours function as a complex multicellular organ composed of both cancer cells and tumour stroma with significant interactive cross-talks [71]. It is thus unsurprising that tumour progression may be driven by molecular alterations in cancer cells as well as the tumour-associated stromal microenvironment [71–74]. GC cells invading into surrounding stroma will thus be confronted with neutrophils [75], carcinoma-associated fibroblasts (CAFs) [72, 74, 76], and a range of bone marrow-derived cells such as mesenchymal stem cells (MSCs) [72, 77, 78] and tumour-associated macrophages (TAMs) [79]. In response, it has been shown that cancer cells generate a variety of growth factors, chemokines and proteases that modulate surrounding stroma to establish a tolerant and contributory stromal environment for tumour progression [71].

### Stromal cells

Stromal cells can heighten the aggressiveness and invasiveness of cancer cells through different molecular signalling pathways. For instance, interactions between neutrophils and MSCs via an IL-6–STAT3 axis lead to neutrophil activation and MSCs differentiation into CAFs, which provide a pro-inflammatory habitat. These stromal cells in turn collaboratively induce angiogenesis and invasiveness of GC cells to stimulate metastatic dissemination [75]. Furthermore, CAFs, derived from bone marrow, and MSCs are frequently enriched during progression into dysplasia. They express cytokine IL-6, glycoprotein Wnt5α, bone morphogenetic protein BMP4, as well as exhibit DNA hypomethylation and induce invasive growth [72]. Additionally, enhanced IL-17B expression in GC tissues leads to MSCs activation and increased migration and stemness, which further accelerates GC cell migration [77]. Interestingly, a recent study showed that MSCs are recruited and reprogrammed in tumour-specific manner. For example, lung cancer cell characteristics are independent of their MSC counterparts while GC cell proliferation, migration and invasion are dependent on the activation of hepatocyte growth factor (HGF)/c-MET signalling pathway specifically induced by HGF from GC-MSCs [78]. Moreover, gene-expression profiling of GC patients has identified a 'stromal-response' expression signature, which is highly enriched in inflammation-, ECM-, cytokine- and growth factor-related proteins. Most of these genes are specifically expressed in the surrounding stroma, but not cancer cells themselves, indicating the important role of stromal cells in promoting GC cell migration and metastasis [79].

### Angiogenesis

Angiogenesis represents a tumour response to the hypoxic and nutrient-deficient environment driven by uncontrolled cellular proliferation and consequent explosive enlargement of tumour bulk [80, 81]. This process is fine-tuned by multiple signalling molecules and pathways in the tumour microenvironment. For example, miR-130a and miR-495 mediated downregulation of RUNX3, a suppressor of tumour angiogenesis, induces the metastatic ability of GC cells [82]. Based on the hypothesis that neovasculature can be formed through sprouting new vessels from existing blood vessels, emerging evidence indicate that tumour-associated angiogenesis can be initiated by cells recruited from the bone marrow or differentiated from putative cancer stem-like cells [81, 83]. Tumour-induced neovascularisation serves to supply sufficient oxygen and nutrients to meet the metabolic needs of uncontrolled tumour growth. Furthermore, studies show that tumour-associated angiogenesis are usually leaky and tortuous with high permeability, which could increase the chance of surrounding tumour cells intravasating into the blood circulation and disseminating to distant sites [12, 81, 84].

### Lymphangiogenesis

Recent studies have shown that the growth of lymphatic vasculature, also known as lymphangiogenesis, either around the tumour or in the sentinel lymph nodes, is associated with increased incidence of lymphatic metastasis [85, 86]. In GC patients, lymph nodes are among top metastatic destinations, and accumulating evidence has shown that LN metastasis predicts GC prognosis [87, 88]. A study reported that the lymphatic vessel density (LVD) within lymph nodes is closely associated with nodal metastasis and malignancy of GC. Concomitantly, GC patients with high LVD showed notably poorer prognosis compared to low-LVD group, suggesting that intranodal lymphangiogenesis is tightly correlated with lymph node metastasis and poor prognosis in GC patients [86]. Mechanistic studies have highlighted the molecular mechanisms underlying the regulation of lymphangiogenesis. For instance, it has been shown that VEGF-C, VEGF-D and VEGFR-3 have an inducive role in promoting lymphangiogenesis in various cancers [85, 89–91], including GC [86, 92, 93]. Using human lymphatic endothelial cells co-cultured with VEGF-C-induced high-lymphangiogenesis GC cell line MKN45 and SGC-7901, the researchers identified several lymphangiogenesis-associated microRNAs such as upregulation of miR-648, miR-5002-3p and downregulation of miR-3178, miR-593-5p, miR-4485 [92]. Rosiglitazone [87], a peroxisome proliferator-activated receptor γ (PPARγ) agonist, has shown promising suppressive effect on lymphangiogenesis by concurrently downregulating the expression of VEGF-C and VEGFR-3 in GC xenograft mice models [93].

Collectively, these findings provide evidence that interactions between cancer cells and the tumour-associated stromal microenvironment could establish a potential positive-feedback loop, which provides substantial contributions to GC progression and metastasis. Accordingly, it is reasonable to hypothesise that tumour malignancy may be suppressed or even reversed by normalising the stromal environment.

## Intravasation into the circulation

During the path of local invasion, cancer cells may encounter blood vessels or lymphatics to facilitate movement towards distant pre-metastatic niches. Alternatively, they may reach and penetrate beyond the serosa to initiate intraperitoneal seeding or direct invasion into neighbouring organs. Here we focus on intravasation which describes the process in which cancer cells gain access into the tumour-associated vasculatures located in the gastric submucosa [10, 43, 93].

Intravasation can be accelerated by molecular alterations that improve the potency of cancer cells in transendothelial invasion. Accumulating evidence has shown the positive correlation among vascular invasion, intratumoral angiogenesis and distant metastasis [94, 95]. For example, the first cloned member of CCN family, Cysteine-rich 61 (Cyr61), was shown to enhance the IL-8-dependent chemotactic migration of GC cells through inducing CXCR1/CXCR2 function, which promotes transendothelial invasion and intravasation [96].

Apart from its role as passive channels for tumour cell dissemination, emerging evidence also illustrated that lymphatic vessels actively stimulate recruitment of tumour cells to lymph nodes, immune regulation and cancer cell survival [85, 89]. The quantity of lymphatic vessels in the vicinity of primary tumours correlates with the rate of lymph node metastasis, and lymphatic metastasis is a key factor for prognosis and tumour staging in majority of cancers [85, 90, 97].

## Intraperitoneal spread after serosal penetration

In addition to distant metastasis, ~10–20% of GC patients were found to harbour peritoneal metastasis that have likely arisen from exfoliated cancer cells through penetration of the gastric serosa [13, 98, 99]. However, this is likely to be an underestimation as intraperitoneal seeding was subsequently found in some who had undergone radical gastrectomy [98]. These microscopic metastases can initially be difficult to identify by imaging or even during surgery, and is only realised when patients present with progressive disease despite curative surgery. The field has yet to identify any molecular alterations that facilitate this pathway.

## Survival within vasculature transition and intraperitoneal environment

Following successful intravasation into the circulation, the disseminating cancer cells, now termed circulating tumour cells (CTCs), must survive the precarious microenvironment en route to new sites of dissemination. The exposure to blood introduces stressors such as haemodynamic shear forces and recognition by the innate immune system. Furthermore, CTCs must also gain the ability to survive in the absence of substratum [100–102].

### Anoikis resistance

The concept of 'anoikis' represents a form of programmed cell death triggered by loss of ECM attachment in epithelial cells [103]. Anoikis is crucial for maintaining epithelial architecture by prohibiting abnormal proliferation in unwanted locations after detachment. Cancer cells are frequently resistant to anoikis, which enable them to survive and thrive even after detachment from its substratum. Anoikis resistance is mechanistically facilitated by cell adhesion molecules, integrins and apoptosis modulators [103, 104], which promotes cell survival and dissemination in the periphery, thereby increasing the possibility of metastatic spread. For example, the peritoneal dissemination of GC cells can be inhibited by Caspase-8-augmented anoikis, which reduced cell survival in vitro and in vivo [105]. Meanwhile, tight junction protein Claudin-1 can induce anoikis resistance through β-catenin-modulated cell–cell adhesion and survival signals [106]. Of interest, RhoA, which belongs to Rho family GTPases, is upregulated in primary GC and its activation has been suggested to be essential for anoikis resistance by eliciting pro-survival responses [4, 107]. Hypoxia-induced ANGPTL4A in GC cells also induces increased resistance to anoikis by activating ANGPTL4A-dependent FAK/Src/PI3K-Akt/ERK pathway, leading to elevated peritoneal metastasis in scirrhous GC cells [108].

### Platelets

Auxiliary pro-metastatic signals exist during intravascular transition in the circulation to aid cancer metastasis. Emerging evidence has shown that the interaction between platelets and cancer cells, more specifically the formation of emboli, are constructive in priming CTCs for intravascular survival [100, 109–111]. On the one hand, the platelet-coated tumour cells can protect them from blood flow shear forces, substratum absence and direct lysis by natural killer cells [112]; on the other hand, their association could also induce EMT in cancer cells [109], enhance adhesion to endothelial cells [111], or even disrupt the function of endothelial barrier, making it more porous for extravasation of cancer cells [101]. One recent study postulated an association between microRNAs and platelets using microRNA microarray analysis of MKN45 cell line. The group identified miR-4670-5p as the most significantly upregulated microRNA that promoted GC cell proliferation and that its proliferation-promoting effects are inhibited by aspirin in vivo [113]. This finding is consistent with five large randomised clinical trials showing that platelet inhibition by low-dose aspirin is beneficial in reducing the incidence of cancer metastasis [114]. Platelet micro-particles (PMP), submicroscopic vesicles shed by activated platelets membrane, are significantly upregulated in GC patients as compared to healthy subjects. Plasma PMP can be used as a platelet activation marker for GC diagnosis and to screen GC patients with increased potential for metastasis [115]. These results indicate that interaction between platelets and CTCs function as intrinsic determinants for distant metastasis through promoting cancer cell survival during intravascular transition, thereby raising the prospect of developing platelet inhibition drugs to aid anti-metastasis therapy.

## Extravasation into 'fertile soil' at distant pre-metastatic niches

Despite the theoretical possibility that CTCs can be deposited at any metastatic niche within or surrounding both circulation systems, clinical observations have shown that certain cancer types have a higher probability of giving rise to metastasis in certain target organ(s) because of exosome-initiated pre-metastatic niches formation. For example, GC tends to form distant metastasis in the liver, peritoneum, lung, bone and lymph nodes [10]. Two hypotheses have postulated the pattern of metastasis tropism: (1) passive transfer, whereby the site of dissemination is dependent on vessel diameter as circulating cancer cells are arrested as they reach the microvasculature, which suggests that the metastatic pattern could be influenced by the layout of circulation systems [116]; (2) active homing, whereby the CTCs have genetically programmed receptor–ligand signalling that have predetermined predilections to target specific organs [6].

### Extravasation

Extravasation represents the exiting of circulating cancer cells out of the vessel lumen to establish new sites of metastasis. There are two recognised forms of extravasation dependent on vessel diameter. Firstly, CTCs with adhesive molecules on the surface can attach to and penetrate the endothelium of the vessel walls irrespective of vessel size [6]. Alternatively, CTCs may be arrested and trapped at the microvasculature due to their relatively larger diameters of 20–30 μm compared to that of around 8 μm [6]. Once trapped, CTCs tend to grow into microcolonies which disrupt the luminal wall and invade into the surrounding tissue environment. Emerging evidence shows that the latter choice is the prevalent pathway by which CTCs grow into a distant metastasis, as single extravasated cancer cells may easily be eliminated by the surrounding microenvironment [117].

Naturally, factors that promote vasculature permeability are associated with increased extravasation. Calponin h1, an actin-binding protein which is mainly expressed in smooth muscle cells, plays a role in stabilising the actin filament system. Calponin h1 deficiency can induce the fragility of blood vessels and peritoneum, leading to the increased incidence of extravasation and tumour metastasis [118]. Accumulating evidence has shown that ANGPTL-4 plays a role in promoting metastasis by inducing the permeability of vasculatures in cancers that metastasise to the lungs [119, 120], and that ANGPTL-4 can increase the frequency of venous invasion. The potential role of ANGPTL-4 in disrupting vascular permeability in promoting GC metastasis requires further investigation [121].

Furthermore, studies focusing on targeting extravasation has led to the discovery of a double anti-angiogenic decoy receptor, double anti-angiogenic protein (DAAP), which simultaneously targets VEGF-A and angiopoietins to block tumour-associated angiogenesis and vascular leakage [122]. Hence this suggests that there is potential for analogues to be developed that can limit primary tumour growth as well as inhibit distant spread.

### Exosome and pre-metastatic niche formation

Exosomes are membranous nanoparticles 40–50 nm in diameter and they can be released by both tumour cells and surrounding stromal cells, which will interact reciprocally to modulate immune responses, remodel tumour microenvironments and facilitate cancer metastases [123–125]. The role of GC-derived exosomes in metastasis has been extensively studied over the years.

GC-derived exosomes can modulate immune responses. For example, GC-derived exosomes can stimulate macrophages to generate a pro-inflammatory microenvironment via activation of nuclear factor κB (NFκB) signalling pathway, resulting in increased cell proliferation and migration [126]. Similarly, GC-derived exosomes enveloped with miR-451 can be translocated to infiltrating T cells and induces mTOR signalling pathway activation, which in turn leads to T-helper 17 (Th17) cells differentiation [127]. Hence, GC-derived exosomes may play important roles in mediating immune surveillance escape.

In terms of tumour microenvironment remodelling and cancer metastasis, mounting evidence indicates that GC-derived exosomes can initiate or accelerate pre-metastatic formation [124, 128, 129]. For example, EGFR-containing exosomes secreted by GC cells can be transported to liver and activate hepatocyte growth factor (HGF), which interacts with c-MET on disseminated GC cells in a paracrine fashion, thereby further promotes their colonisation and proliferation [130]. In addition, another study demonstrated that GC-derived exosomes can bolster pre-metastatic niche formation in peritoneum by inducing fibrosis and the disruption of mesothelium, which originally functions as a protective barrier to restrain peritoneal metastasis [131]. Similarly, GC-derived exosomes can promote expression of adhesion-related molecules, such as fibronectin 1 (FN 1) and laminin gamma 1 (LAMC 1), in mesothelial cells, which result in a favourable microenvironment for disseminating cancer cells to colonise and initiate metastasis [132].

Nevertheless, researchers are utilising the unique features of exosomes for drug delivery. Exosomes are loaded with drugs or siRNA to target the tumour regions [133, 134]. Exosomes loaded with HGF siRNAs have shown promising efficacy in inhibiting tumour growth, migration and angiogenesis in vitro and in vivo [133]. Exosomes isolated from heat stress-treated malignant ascites of GC patients showed elevated immunogenicity and might be employed as a cancer vaccine. Such exosomes can induce dendritic cell maturation and stimulate a tumour-specific cytotoxic T lymphocyte response [135].

Despite intensive efforts, limitations still exist in the study of exosomes as they are mainly restricted to in vitro co-culture or in vivo injection using labelled-exosomes, which is markedly different from their physiological location and concentration [123]. These concerns accentuate the necessity of developing novel models to overcome the limitations in exosome studies.

## Colonisation and proliferation reactivation into clinical detectable metastases

Given the divergent microenvironment of the metastasised sites from that of the stomach, successfully extravasated cells need to adapt to the foreign microenvironments in order to survive and colonise. Currently, there are two universally acknowledged mechanisms by which cancer cells adapt to their new microenvironment: (1) cell autonomous programmes and (2) non-autonomous programmes [6].

For cell autonomous programmes, disseminating tumour cells (DTCs) acquire molecular alterations to increase their colonising ability. Colonising abilities are normally evaluated by detecting pulmonary metastases after intravenous (IV) injection in immune-deficient mice. For example, ectopic expression of RUNX3 repressed lung colonisation of GC cells in nude mice [28]. Similarly, the silencing of IL-32 in GC cells inhibited cell motility, invasion and lung colonisation in severe combined immunodeficiency (SCID) mice [136]. In the case of peritoneal metastasis after serosa penetration, increased expression of connexin 43 (Cx43) in GC cells exfoliated into peritoneal cavity was found to enhance their heterocellular gap-junctional intercellular communication (GJIC) with peritoneal mesothelial cells, which in turn mediated heterocellular gap junction and accelerated the infiltration of GC cells into peritoneal mesothelium for further colonisation [137]. This finding provides implications for further studies on GC cells seeded onto the lining mesothelial layer. Another mechanism is based on ligand-receptor interaction. For example, the expression of stromal cell-derived factor-1, together with its sole interactive receptor CXCR4, correlated with increased probability of lymph node and liver metastases [138]. For non-autonomous programmes, certain organ sites provide supportive niches which better facilitate the survival of DTCs. A recent retrospective study found that patients with STAT3 activation in cancer cell-free lymph nodes demonstrated higher rate of metastasis and poorer prognosis, which implicated the possibility of p-STAT3-induced pre-metastatic niches in lymph nodes [139]. Indeed, STAT3 blockade in myeloid cells abrogated the formation of pre-metastatic niches [140]. Moreover, the inflammatory cytokine tumour necrosis factor-α (TNFα) has been reported to induce morphological changes of mesothelial cells and regulate interactions between peritoneal mesothelial cells and DTCs, which in turn promotes peritoneal metastasis of GC cells in the intraperitoneal (IP) injected mouse model [141]. Hence, cytokines and chemokines also play a role in the shaping of pre-metastatic niches for GC peritoneal metastasis. Collectively, both autonomous and non-autonomous programmes promote cancer cells’ colonisation in pre-metastatic niches.

However, clinical observations showed that relapses are often detected long after removal of the primary tumour, spanning from months to years even when there was no previous evidence of metastasis [142]. This implies that these patients already carry DTCs in the body, where those DTCs remain dormant in two modes: (1) cellular dormancy and (2) tumour mass dormancy [143]. For example, in some GC patients, dormant DTCs can be detected harbouring inside bone marrow and they eventually develop into detectable metastasis in brain after 10 years, illustrating that dormant DTCs derived from GC retained both metastatic and growth ability for long periods of time [143, 144].

Latency represents a state in which metastatic cancer cells undergo proliferative quiescent in order to escape from immune clearance, attack from the new microenvironment and the surrounding growth inhibitory signals. They remain latent until certain, currently unknown factors re-activate their proliferative potency. At present, our knowledge of the underlying mechanisms of latent metastasis is limited due to the lack of mouse models that faithfully recapitulates the metastatic process and microenvironment.

## Conclusion and perspective

Over the past decades, research progress on GC metastasis-related molecular alterations has provided valuable knowledge for deciphering this complex biological phenomenon. Although by no means comprehensive, we have rapidly gained an appreciation for the importance of stromal cells and the microenvironment. Nevertheless, due to the complexity and systemic nature of metastasis, a number of fundamental questions concerning the mechanisms of GC metastasis remain unanswered.

The major hurdle in the study of tumour metastasis is the lack of a mouse model with a competent immune system that can perfectly mimic the entire metastatic cascade. Therefore, this bottleneck imposes restrictions on in-depth study of the latter stages in the GC metastatic cascade. Attempts to establish a better metastatic mouse model have recently achieved intriguing progress, such as the implementing genome-wide or high-throughput screening approaches into immune-competent mice for identification of novel regulators of metastases [145, 146]. In this way, researchers can evaluate both tumour-cell-intrinsic (molecular manipulation of cancer cells) and tumour-cell extrinsic factors (tumour microenvironment of genetically engineered mice or drug treated mice) that modulates the metastasis cascade. Interestingly, a recent study used vascular endothelial growth factor receptor (VEGFR3) as an 'lymphorecporter' and established a novel mouse model that allows whole-body imaging of lymphovascular niches, which shed new lights on pre-metastatic niches [147]. These innovative technologies can all be considered as tools for future GC metastasis study.

Metastatic cancer cells that have successfully intravasated into the circulation system can survive and extravasate efficiently (>80%) [145]. This phenomenon suggests that effective mechanisms exist to protect CTCs from being eliminated during the transition. Mounting evidence has shown the correlations between neutrophil/lymphocyte ratio (NLR) and GC patients outcome that high NLR predicts poor prognosis and survival status [148–150]. However, few research has done on the mechanism by which neutrophils interact with CTCs in the circulation system to promote metastasis progression. Meanwhile, the role of other tumour-infiltrating immune cells, such as TAMs, natural killer (NK) cells, CAFs, also deserves further investigation.

Mechanistically, apart from the widely reported function of MMPs in the degradation of ECM, recent studies have revealed alternative roles of MMPs in metastasis, such as regulation of growth signals, apoptosis, tumour vasculature, inflammation and non-proteolytic functions [42]. Moreover, long noncoding RNAs (lncRNAs) are also gaining attention, since emerging data indicate that the deregulation of lncRNAs might contribute to tumour metastasis [151–154]. Apart from cell intrinsic alterations that contribute to GC metastasis, tumour-derived exosomes also showed great influences on inducing pre-metastatic niches [128]. These potential targets also deserve further exploration.

We hope that our understanding of the evolution of cancer metastasis continues to excel at this impressive pace, and that some of these findings will be translated into clinical use, especially in light of the current proportion of patients with metastatic GC.

## Disclaimer

Neither the submitted paper nor any similar paper, in whole or in part, other than an abstract or preliminary communication, has been submitted to or published in any other primary scientific journal. All the authors are aware of and agree to the content of the paper, copyright assignment and authorship responsibility.
